# Among the Out-Patients

**Published:** 1886-12-11

**Authors:** 


### Among the Out-Patients.

By One of the Crowd.

A New Country.

It has often been a matter of surprise to me that none of the clever gentlemen who write for the daily press have ever thought of exploiting the hospital out-patient. The casual wards of the workhouse, the slums, the common lodging-houses, the pawnbrokers' shops, have all formed the subject of more or less interesting articles, but, so far, the steps of the men who wrote what may be called, for want of a better name, *genre* articles in the newspapers, have never been directed inside any one of the great institutions which, between them, attend to the ailments of some 40,000 Londoners every year. Perhaps the attempt has been made, and has failed; although, on second thought, this is improbable, seeing that, to paraphrase a well-known quotation, "In the bright lexicon of journalism there's no such word as 'can't.'" It must be confessed that, on first acquaintance, the hospital out-patient is not a very interesting-looking nor a very communicative personage. At least, that was my experience when, in my capacity as representative of The Hospital, I paid my first visit to the waiting-room at Guy's. I chose Guy's, not out of any respect for the injunction *seniores priores*, but on account of the very peculiar, and to my mind excellent, system which prevails there, and which was recently dealt with in these columns by Dr. Steele, the Superintendent, to whose inception, I should add, it is mainly due. One might, I thought, reasonably expect to find here the "hupper sukkles" of out-patients, but in truth they differed little, if at all, from their prototypes at "Barts" or St. Thomas's.

Payments and Patients.

The inference seems to be that, speaking generally, out-patients as a class can very well afford to pay threepence for their weekly supply of medicine, and if they can't—well, there is no degradation, certainly no taint of pauperism, involved in that application to the Charity Organisation Society which is required by the hospital authorities. I am not quite sure, either, whether the system has not a moral effect upon the people more satisfactory even than its results to the finances of the Charity. I asked one old lady who had accompanied her grand-daughter to the medical side, what she thought of it. "Well, you see," said she, "I'm a widow, and finds it not so easy to get along, especially in the winter, which *is* beginning to be trying to me; but there's only Lizzie here (referring to her grand-daughter), and she'll soon be able to earn something. When you pays your threepence a week you feels that it ain't quite a dole like; and lor! look what good this place does. Besides," she added, with a knowing nod of the head, "threepence no more pays for what you get than——" A comparison failed her, and the conversation ended. These sentiments are not improbably those almost universally held by the out-patients, for barely six per cent. during the whole three years the system has been in operation have declared their inability to pay, and even of these only about a third have been really proved to be fit subjects for the remission of the fee.

Out-Patients as a Class.

And yet Heaven knows that the district of which Guy's is the centre is poor enough, as any-one may see who takes even a casual walk through the Borough. And the patients who come from a distance are not much better off.

Cheap tram fares are a blessing to these latter, and they may be seen in considerable numbers—distinguished always by the neck of a bottle protruding from a wicker-basket—getting off the cars as they stop at St. George's Church. The crowds I have seen in the two large waiting-rooms at Guy's have impressed me chiefly by their distinct unlikeness to the many other conditions of men and women with which I have been thrown into contact. These are not faces which tell of despair, or of any actual want of modest comfort. They express rather the conviction that life has been, is, and must be a struggle for bread and butter. There is much need for dozens of Kyrle Societies, and they might all labour for the next twenty years in London without ever treading on one another's toes. The respectable working classes—not the highly paid skilled artisan, I mean, but the people who can just keep heads above water—the people, in fact, who form the vast proportion of the population of this Metropolis—have a lot in life less enviable than that of any other class with which I am acquainted. It is all drudgery. Existence for them is a succession of sombre hues. Their life is by no means a *chiaro-oscura*, but rather an arrangement in sad brown or dull grey, with only a tiny speck of bright colour here and there. This, at least, is the impression which forced itself on my mind as I watched the hundred or two men and women gathered together in each of the large rooms where the medical and surgical out-patients of Guy's Hospital "wait their turn."

**Relaxation and Work.**

A neat, bustling little woman in the first-named department was knitting away at a pair of little stockings as if her life depended upon her getting them finished before she was called into the inner hall, where the patients range themselves by twenty or thirty at a time, and out of which the physicians' consulting-rooms open. The other women all about were either doing nothing or reading the halfpenny or penny "novelettes," the weekly publication of which, by the tens of thousands, is one of the first-fruits of the Education Act. I was somewhat chary of interrupting my busy friend, but when I mustered up courage to do so, it was easy to see that this "trash," as she called the *brochures* in question, was her pet aversion. "I've no patience with girls nowadays," she declared, with emphasis. "Look at this girl," she continued, in a tone which attracted the attention of the damsel referred to, a be-draggled young woman, not unprepossessing in appearance, and adorned with cheap and unsubstantial finery from top to toe. "Look at this girl! Hadn't she better bring some work to do whilst she is waiting, instead of filling her head with such rubbish—ay! and worse than rubbish, very often?" The "girl"—who wore a wedding-ring, by the way, and was quite two or three-and-twenty — tossed her head scornfully. "She had to work hard all day," she said, "and 'twas a pity she couldn't have a little amusement now and then." I perceived I had inadvertently raised a discussion which might have become bitter, and was bound to be inconclusive. I was commencing to lay the blame at the door of the industrious woman with the loud voice, when, fortunately for all of us, a move took place, and the "girl" went into the inner hall.

**Ought She to have been there?**

The noise had attracted the attention of another out-patient, the sight of whom diverted my thoughts into quite another channel. This person was substantially, not to say richly, dressed; the black satin mantle, with its fur trimming, was nearly new, and had cost, I am sure, as much as all the other women who sat on the same bench could have scraped together by the sale of all their worldly effects. She accompanied a child, who was also comfortably clad, and the discussion which I had been the means of raising caused her to turn her head round. She gazed at her fellow-patients with a look of scorn upon her face, and gathered herself together as if to avoid contamination. I could not help asking myself two questions: first, how she could reconcile it with her conscience to attend here and share in benefits which were never intended for persons in her class of life; and secondly, how she had managed to see the doctor in the first instance: for she was not an original patient, as my knowledge of the regulations of the hospital enabled me to discover. There are some people whose dearest pleasure it is to "get something for nothing," and I can quite understand that on the occasion of her first visit she was careful to attire herself in a less ostentatious fashion.

**Circumstances and Clothes.**

That this is not unfrequently done, an incident which happened some years ago in a London hospital will show. A man of humble position, but careful and scrupulously neat in his attire, journeyed from a northern town to the metropolis on purpose to see a celebrated doctor who sat at the institution I am referring to. His worldly position and his numerous family made it quite impossible that he could pay the fee which the specialist would demand from an ordinary patient, but when he went to the hospital and was called in to the presence of the doctor, that gentleman looked at him severely, and said: "Sir, hospitals are not instituted for persons such as you," and called for the next patient. Emerging somewhat crestfallen into the waiting-hall, a man with whom he had been talking, asked, "How did you get on?" and having heard the other's narration, coolly observed: "you must be a greenhorn to dress yourself in your best to come here."

---

Medicinal Boots.—A Pennsylvanian shoemaker advertises "Medicinal Boots." What they are, we have been unable to guess, but the funny man on our staff suspects they must have some particular virtue in the *heal.*

